# Chromatographic analysis of ponatinib and its impurities: method development, validation, and identification of new degradation product

**DOI:** 10.3389/fchem.2024.1487108

**Published:** 2024-11-12

**Authors:** Jing Wang, Yonghong Zhu, Jisu Qin, Wenyi Wu, Rongrong Huang, Liangliang Cai

**Affiliations:** ^1^ Department of Pharmacy, Affiliated Hospital of Nantong University, Nantong, China; ^2^ Department of Pharmacy, Affiliated Nantong Hospital of Shanghai University (The Sixth People’s Hospital of Nantong), Nantong, China; ^3^ Department of Pharmacy, Nantong University, Nantong, China; ^4^ Department of quality inspection, Sinopharm holding Nantong Ltd., Nantong, China

**Keywords:** ponatinib, liquid chromatography, novel compound, method development, method validation, related substances

## Abstract

**Background:**

Ponatinib, a third-generation tyrosine kinase inhibitor, is employed in the management of adult chronic myeloid leukemia. Nevertheless, the presence of process impurities and degradation impurities linked to ponatinib may potentially influence its effectiveness and safety. Therefore, the objective of this research was to establish a robust liquid chromatography method and systematically validate it for the detection of substances related to ponatinib.

**Methods:**

The separation of ponatinib and its impurities was conducted using an Agilent 5HC-C_18_ chromatographic column (4.6 mm × 250 mm, 5 μm). The mobile phase A comprised a mixture of water and acetonitrile in a 9:1 ratio, with an aqueous solution of pH 2.4 containing 2 mM potassium dihydrogen phosphate and 0.4% triethylamine. Mobile phase B, consisting of acetonitrile, was eluted in a gradient fashion. The flow rate was set at 1.0 mL/min, detection wavelength at 250 nm, column temperature at 40°C, and injection volume at 10 μL.

**Results:**

The method demonstrated high specificity, sensitivity, solution stability, linearity, precision, accuracy, and robustness. Additionally, this research unveiled a novel compound, imp-B, generated via the oxidative degradation of ponatinib. The molecular structure of the newly discovered product was elucidated through the utilization of nuclear magnetic resonance (NMR) and high-resolution mass spectrometry (HRMS).

**Conclusion:**

In conclusion, the chromatographic method developed in this study has the potential to be utilized for the detection of ponatinib and its impurities, thereby offering significant insights for quality assessment in ponatinib research.

## 1 Introduction

Chronic myeloid leukemia (CML) is a hematological malignancy characterized by the presence of a distinct genetic aberration referred to as the Philadelphia (Ph) chromosome (Hochhaus et al., 2020; Spagnuolo et al., 2019). This genetic aberration arises from a reciprocal translocation between chromosome 9 and chromosome 22 (Du et al., 2022; Ma et al., 2022). This genetic aberration gives rise to the formation of a chimeric fusion protein called BCR-ABL1, which exhibits uncontrolled tyrosine kinase activity (Tanaka et al., 2022). The presence of this protein induces dysregulation of signaling pathways that promote cell survival and proliferation in leukemic cells.

Tyrosine kinases are enzymes that use ATP as a phosphate donor to catalyze the phosphorylation of specific tyrosine residues in target proteins (Arshad et al., 2023; Peng and Fu, 2023). These kinases play a pivotal role in regulating a wide range of physiological and biochemical processes, including cellular growth, differentiation, and programmed cell death (apoptosis) (Alanazi et al., 2022; Mondelo-Macía et al., 2020; Richardson et al., 2023). Dysregulated expression of tyrosine kinases often results in the loss of control over cellular proliferation, consequently promoting tumorigenesis (Katayama, 2018). In response, specific tyrosine kinase inhibitors have been developed to selectively inhibit the activity of the BCR-ABL1 tyrosine kinase domain, thereby improving clinical outcomes and prognosis for patients with chronic myeloid leukemia (CML) (Hou et al., 2021; Kantarjian et al., 2021; Lin et al., 2020).

Ponatinib, a third-generation tyrosine kinase inhibitor (TKI), was approved by the Food and Drug Administration (FDA) in 2012 for treating chronic myeloid leukemia (CML) and Philadelphia chromosome-positive acute lymphoblastic leukemia (Ph+ALL) in adults (Cortes et al., 2018; Jain et al., 2019; Massaro, Molica and Breccia, 2018; Pavlovsky, et al., 2019). Previous research on ponatinib primarily focused on the drug’s safety and efficacy in clinical treatment, along with its plasma drug concentration and metabolites (Abumiya, Miura and Takahashi, 2018; Cortes et al., 2018; Kidoguchi et al., 2021; Massaro et al., 2018; Yasu et al., 2018). Recent studies have specifically examined the degradation products generated during destructive testing of ponatinib (Golla et al., 2023). These degradation products are significant constituents of ponatinib-related substances. It is widely recognized that monitoring the presence of related substances is crucial in ensuring the safety, effectiveness, and overall quality of drugs (Ni et al., 2022; Pawar et al., 2024). However, to the best of our knowledge, research on the detection of ponatinib-related substances remains limited. Hence, it is necessary to develop a method for detecting ponatinib -related substances.

This research has successfully established and implemented a validated reversed-phase high-performance liquid chromatography (RP-HPLC) technique for the detecting ponatinib -related substances. The method demonstrates simplicity, sensitivity, accuracy, and robustness. In particular, it effectively separates compounds related to ponatinib, such as impurities derived from the production procedure and degradation products (imp-A, -B, -C). Following that, a thorough evaluation was performed to determine specificity, sensitivity, solution stability, linearity, precision, accuracy, and robustness. Parameters such as limits of quantification (LOQ), limits of detection (LOD), linearity, and recovery of the RP-HPLC method were evaluated. Furthermore, in the study, we discovered a novel compound designated imp-B, originating from the oxidative degradation of ponatinib. The structure of this compound was successfully determined using nuclear magnetic resonance (NMR) and high-resolution mass spectrometry (HRMS) techniques. Overall, the established RP-HPLC method represents a novel approach to advancing process development and quality evaluation of ponatinib.

## 2 Materials and methods

### 2.1 Chemicals and reagents

The compound ponatinib was obtained from MCE China (Shanghai, China). imp-A, and imp-C were obtained from Shanghai Macklin Biochemical (Shanghai, China), while imp-B was homemade by the laboratory of the authors. Merck (Darmstadt, Germany) provided HPLC-grade ACN and methanol (MeOH). Other analytical grade chemical reagents were purchased from China National Pharmaceutical Group Chemical Reagent Co., Ltd., (Beijing, China).

### 2.2 Instruments

For this investigation, a RP-HPLC device (Agilent 1200, Agilent, United States) and a UV-visible spectrophotometer (Cary100, Varian, United States) were utilized. Furthermore, a Milli-Q water purification system was employed.

### 2.3 HPLC conditions

For the separation of ponatinib and its related impurities, a 5HC-C_18_ column from Agilent (4.6 mm × 250 mm, 5 μm) was employed. Mobile phase A consisted of a water and acetonitrile (ACN) blend in a 9:1 v/v ratio. The aqueous solution contained 2 mM potassium dihydrogen phosphate solution (KH_2_PO_4_) and 0.4% triethylamine. Phosphoric acid adjusted the pH to 2.4. ACN was the only component of mobile phase B. The gradient elution was conducted as follows: mobile phase B maintained a constant concentration of 16% from 0 to 2 min, followed by an increase in concentration from 16% to 30% from 2 to 22 min. Subsequently, the concentration of mobile phase B further increased from 30% to 34% from 22 to 32 min. From 32 to 35 min, the concentration of mobile phase B increased to 55%, remaining constant until 42 min. Between 42 and 42.1 min, the concentration of mobile phase B decreased from 55% to 16%. Finally, from 42.1 to 50 min, mobile phase B maintained a constant concentration of 16%. The wavelength used for UV detection was 250 nm, with a flow rate of 1.0 mL/min and a sample volume of 10 µL.

### 2.4 Preparation and characterization of imp-B

To prepare imp-B, 200 mg of ponatinib was weighed and transferred into a 100 mL flask. Methanol (20 mL) was added to fully dissolve the powder. Subsequently, 30 mL of a 30% H_2_O_2_ solution was added, and the mixture underwent oxidative degradation. The reaction mixture was stirred at room temperature for 20 h before terminating the reaction with manganese dioxide. After centrifugation, the supernatant was decanted, and the crude product was obtained by freeze drying.

The imp-B crude product was purified using a preparative column (Agilent Eclipse XDB-C_18_, 250 mm × 9.4 mm, 5 μm). The mobile phase consisted of a 75% methanol-water solution. The elution time was 6 min, and the flow rate was set at 4 mL/min.

To determine the structure of imp-B, nuclear magnetic resonance (^1^H NMR, ^13^C NMR and 2D NMR) and high-resolution mass spectrometry (HRMS) were employed for analysis. The procedure is as follows: dissolve 10 mg of imp-B or 20 mg of imp-B in DMSO-d6 for ^1^H NMR, ^13^C NMR and 2D NMR analysis. Prior to mass spectrometry analysis, the imp-B sample was diluted to a concentration of 100 ng/mL with a 50% methanol-water solution before being injected into the mass spectrometer.

### 2.5 Preparation of stock solution

#### 2.5.1 Preparation of ponatinib stock solution

To prepare a stock solution of ponatinib, approximately 10 mg of ponatinib should be weighed accurately and placed in a 20 mL volumetric flask. Then, 10 mL of 50% methanol solution is added, and the mixture is sonicated to dissolve the ponatinib. The solution is then diluted to the mark with a 50% methanol solution to obtain a stock solution with a concentration of approximately 0.5 mg/mL.

#### 2.5.2 Preparation of ponatinib-related substances stock solution

To prepare standard stock solutions of known impurities (imp-A, -B, -C), 10 mg of each impurity must be accurately weighed and transferred into separate 20 mL volumetric flasks. A mixed solution of methanol and water, with a volume ratio of 50:50 (v/v), is then added to each flask, followed by ultrasonic treatment to ensure complete dissolution of the impurities. The flasks are then filled to the mark with the mixed solution of methanol and water to achieve impurity standard stock solutions with a concentration of approximately 0.5 mg/mL.

### 2.6 Preparation of mixed solutions and system suitability solutions

To formulate a composite solution of ponatinib bulk drug and its related substances, approximately 10 mg of the ponatinib bulk drug was carefully measured and transferred into a 20 mL volumetric flask. Subsequently, 0.2 mL of reference standard stock solutions containing imp-A, imp-B, and imp -C were introduced. Following this, the solution was further diluted to a final volume of 20 mL by combining equal volumes of methanol and water in a proportion of 50:50 (v/v).

The system suitability solution was prepared by combining equal volumes of acidic and oxidative degradation solutions. The specific steps are as follows: Initially, accurately weigh 10 mg of ponatinib and transfer it into a 20 mL volumetric flask. Subsequently, add 10 mL of methanol to the flask to dissolve the ponatinib. Following this, add 5 mL of 1 M hydrochloric acid solution to the mixture and maintain the reaction at 70°C for 12 h. Upon completion of the reaction, neutralize the solution by adjusting the pH to 7 with 1 M sodium hydroxide. Lastly, dilute the reaction mixture to 20 mL with a 50:50 mixture of methanol (MeOH) and water (H_2_O) (v/v).

A 10 mg measurement of ponatinib bulk drug was accurately added to a 20 mL volumetric bottle and dissolved in 10 mL of methanol. It was then combined with a 1 M hydrochloric acid solution and reacted at 70°C for 12 h. Subsequently, it was diluted to a total volume of 20 mL using a mixture of MeOH and H_2_O in a 50/50 ratio (v/v). Likewise, an additional 10 mg of ponatinib was weighed and then mixed with 10 mL of methanol. Subsequently, it was introduced into a 10 mL solution of H_2_O_2_ (3%) and underwent a reaction at ambient temperature for a duration of 20 h. After the reaction was completed, any residual H_2_O_2_ was decomposed using MnO_2_. The MnO_2_ was then separated through filtration, and the resulting solution was subjected to freeze-drying. Ultimately, the oxidative degradation product was isolated and dissolved in a 50:50 (v/v) mixture of methanol (MeOH) and water (H_2_O).

### 2.7 Preparation of the sample solution

To prepare the sample solution, accurately weigh approximately 10 mg of ponatinib bulk drug and dissolve it in 20 mL of a 50:50 methanol-water solution (v/v). This procedure results in a sample solution with an estimated concentration of 0.5 mg/mL.

### 2.8 Method validation

#### 2.8.1 Specificity

To establish RP-HPLC, it is necessary to fully separate ponatinib from its associated compounds. Consequently, the evaluation of the method’s specificity was conducted. According to the chromatographic conditions under Section 2.3, solvents, system suitability solutions, and mixed impurity solutions were injected separately.

#### 2.8.2 Forced degradation experiments

For the degradation test, approximately 10 mg of ponatinib was accurately weighed and transferred into a 20 mL volumetric flask. It was then dissolved in methanol and exposed to various degradation conditions: acid and alkali hydrolysis, oxidation, photolysis, and heat degradation.

The acid degradation test includes treating the ponatinib solution with 1 mol/L HCl for 5 days at 60°C, while the alkaline degradation test involves treatment with 1 mol/L NaOH for 7 h at 60°C. The oxidation degradation test involves treating the ponatinib solution with a 3% H_2_O_2_ solution for 2 h.

The ponatinib sample underwent heat degradation at 150°C for 6 days, and light degradation was induced by exposing it to an LED tube with an intensity of 4,500 lx for 20 days. The volume was adjusted to 20 mL using a MeOH/H_2_O (v/v 1:1) mixture, and analysis followed the instructions in Section 2.3.

#### 2.8.3 Sensitivity

The sensitivity of the detection method was assessed by determining the LOD and LOQ. Initially, the ponatinib standard stock solution and stock solutions of related substances were sequentially diluted and injected into the sample as per the procedure outlined in Section 2.3. Subsequently, the signal-to-noise ratio (S/N) was calculated, and the concentrations corresponding to S/N ratios of 3:1 and 10:1 were determined as the LOD and LOQ, respectively.

#### 2.8.4 Stability of the solution

For the stability study, the ponatinib sample solution was analyzed at specific time intervals ranging from 0 to 24 h (0, 1, 2, 4, 6, 8, 12, and 24 h). To assess the sample solution’s stability, changes in impurity quantities, maximum concentrations of individual impurities, and overall impurity content were compared.

#### 2.8.5 Linearity and range

The concentration of the provided sample solution of ponatinib (0.5 mg/mL) was defined as 100%. An examination was conducted to determine the linear correlation between ponatinib and its impurities in the concentration range from the quantitative limit to 2.0%. The standard stock solution of ponatinib and its known impurities (imp- A, -B and-C) was diluted using a 50% methanol-water solution to obtain various test solution concentrations, which were then analyzed through injection. A standard curve was generated with concentration on the *X*-axis and peak area on the *Y*-axis to calculate the regression equation.

#### 2.8.6 Precision and repeatability

To assess the accuracy of the instrument, the mixed impurity solution mentioned was injected six times consecutively, employing the high-performance liquid chromatography conditions outlined in Section 2.3. Simultaneous recording of chromatograms was performed, followed by calculation of the RSD values for both the retention time and peak area.

To assess the repeatability of this method, six mixed sample solutions were prepared following the procedure outlined in Section 2.6. Subsequently, injection detection was performed using the chromatographic conditions specified in Section 2.3, and the chromatograms were recorded.

#### 2.8.7 Accuracy

The investigation focused on the recovery rates of ponatinib-associated compounds at three different levels: 50%, 100%, and 150%. The concentration of the related substance at the 100% level was 1 μg/mL. To achieve these concentrations, varying volumes of impurity stock solutions were added to the ponatinib sample solution. Each concentration sample was prepared in triplicate. Subsequently, the samples were injected, and the recovery calculation was conducted.

#### 2.8.8 Robustness

To assess the method’s robustness, we identified the suitable solution of the system across different chromatographic conditions. Chromatographic parameters encompassed the initial mobile phase A-B ratio, detection wavelength, column temperature, flow rate, mobile phase pH, and column type. Table 3 outlines the specific robustness criteria.

## 3 Results

### 3.1 Method development

**FIGURE 1:**
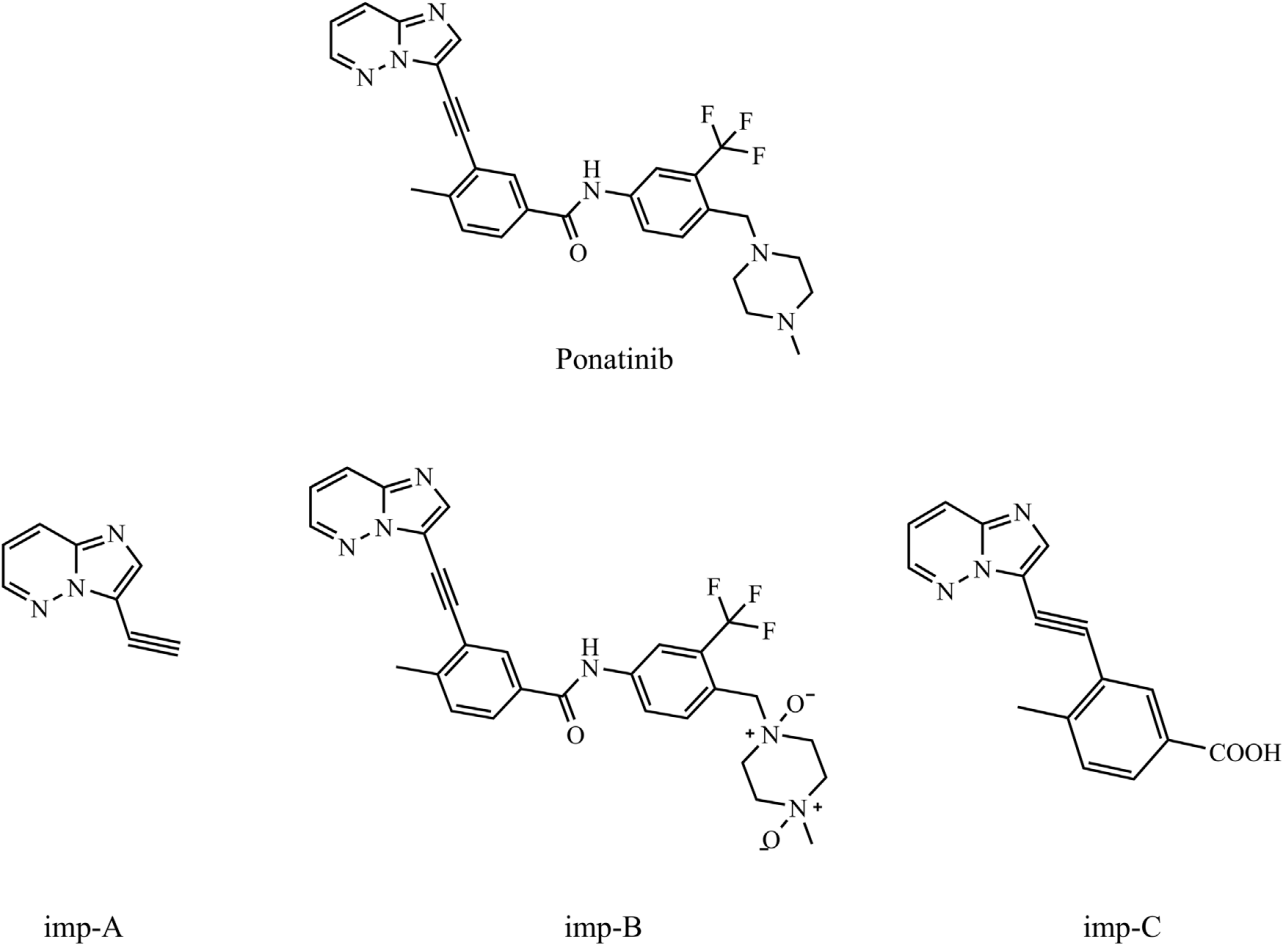
Chemical structures of ponatinib, imp-A, imp-B and imp-C.

The synthesis of ponatinib, a potent TKI inhibitor, follows the pathway of patent W020110539338 as depicted in Supplementary Figure S1. Various starting materials and intermediates are utilized in the synthetic pathway of ponatinib. Forced degradation tests revealed three primary compounds associated with ponatinib: imp-A, imp-B, and imp-C. The chemical structures of ponatinib, imp-A, imp-B, and imp-C are depicted in Figure 1. imp-A is a process-related impurity, whereas imp-C serves not only as a process impurity but also as an alkaline degradation product of ponatinib. Furthermore, imp-B is a novel compound resulting from the oxidative degradation of ponatinib. Its structure was unequivocally established using nuclear magnetic resonance and mass spectrometry. A method employing reversed phase high performance liquid chromatography (RP-HPLC) was developed to determine the concentrations of ponatinib-related substance.

To optimize the method, we systematically investigated the effects of solvent, detection wavelength, mobile phase composition, and elution method on sample separation. To choose an appropriate solvent, ponatinib was dissolved in methanol (MeOH) and acetonitrile (ACN). The findings indicated that ponatinib exhibited low solubility in ACN but was completely soluble in MeOH. Consequently, we conducted additional research on ponatinib in a 1:1 MeOH/H_2_O (v/v) solution and observed that it can dissolve at a concentration of 0.5 mg/mL without any evidence of turbidity. Subsequently, we investigated the influence of the MeOH/H_2_O (v/v 1:1) solvent on the stability, peak shape, and resolution of ponatinib. The results demonstrated that the use of MeOH/H_2_O (v/v 1:1) as the solvent did not result in any significant changes in the impurities of ponatinib over a 72-h period. Additionally, the symmetry factor of the ponatinib peak was 1.12 and the minimum resolution between it and nearby impurity peaks was greater than 1.5. Consequently, MeOH/H_2_O (v/v 1:1) was ultimately selected as the solvent for this study.

To determine the optimal absorption wavelength, a solution of ponatinib standard stock and its associated compounds were diluted by a factor of 50 and then analyzed using UV-VIS scanning within the 200–400 nm range. Supplementary Figure S2 displays the acquired ultraviolet spectra, which unveil significant absorption around 250 nm for ponatinib and its associated compounds. Therefore, the wavelength of 250 nm was selected for detection in this research.

**FIGURE 2:**
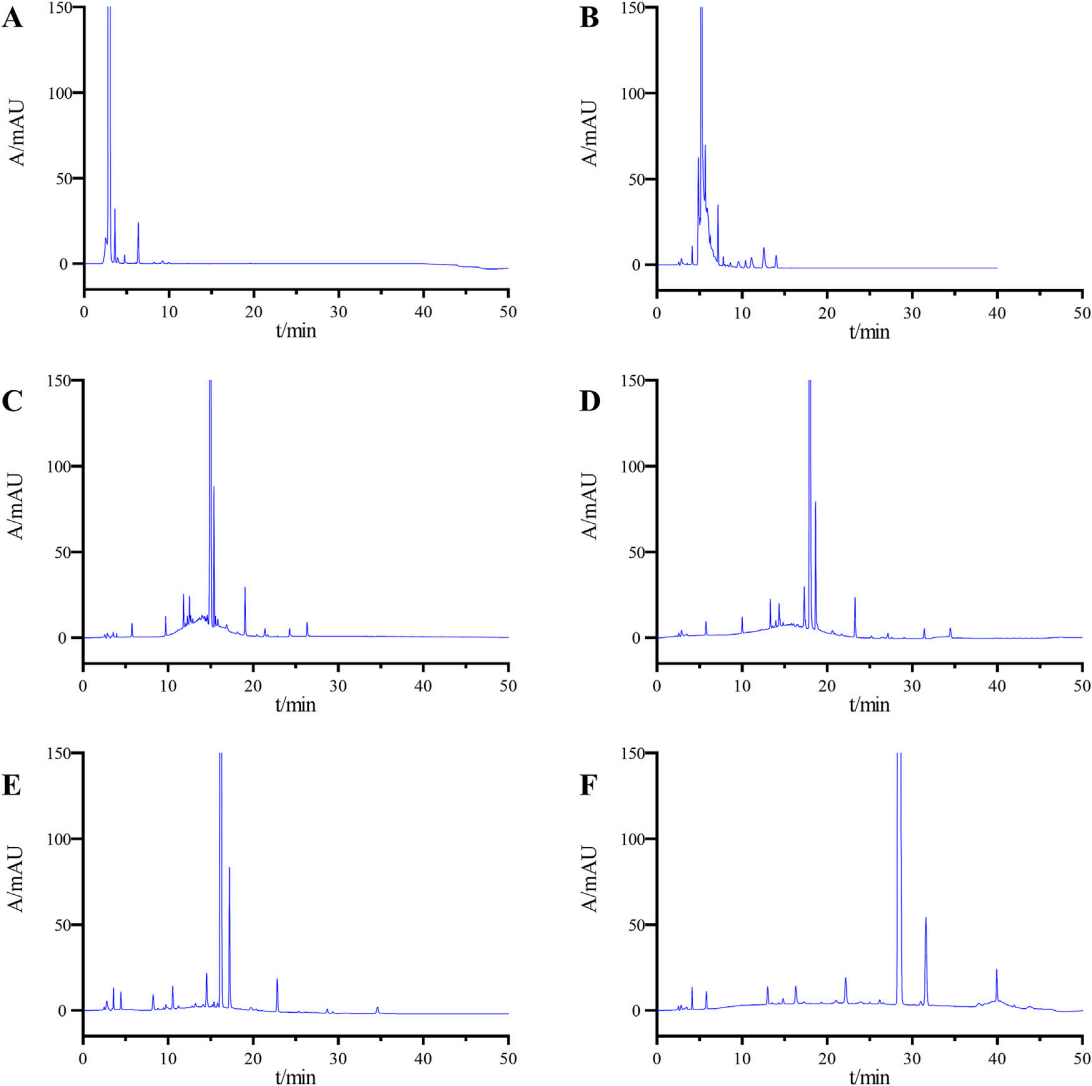
Chromatograms obtained during the optimization of the LC conditions: **(A)** ACN-H_2_O (80:20), **(B)** ACN-H_2_O (60:40), **(C)** gradient elution Condition 1, **(D)** gradient elution Condition 2, **(E)** gradient elution Condition 3, and **(F)** final determined gradient elution condition.

Due to the significant presence of difficult-to-separate impurities in the system suitability solution, this solution was chosen for method development. Initially, different proportions of mobile phase B to mobile phase A (80:20; 60:40) were employed for isocratic elution. The results indicated that the impurities overlapped on the chromatogram, with a limited number of impurities, and the separation degrees both among impurities and between the impurities and the main peak failed to meet the requirements (Figures 2A, B).

Owing to the number of impurities in the system suitability solution, achieving separation of individual impurities using isocratic elution proves challenging. Consequently, the adoption of a gradient elution method was deemed suitable. Initially, gradient condition 1 (0∼2 min, 10%B→10%B; 2∼20 min, 10%B→90%B; 20∼30 min, 90%B→90%B; 30∼30.1 min, 90%B→10%B; 30.1∼40 min, 10%B→10%B) was utilized for analysis, as depicted in Figure 2C. The minimum separation degree between impurities was 1.15, and between impurities and the main peak was 1.85, which did not meet the specified requirements. Subsequently, the gradient rate was optimized by decreasing the rate of change. Sample analysis was conducted using gradient conditions 2 (0∼2 min, 10%B→10%B; 2∼30 min, 10%B→90%B; 30∼35 min, 90%B→90%B; 35∼35.1 min, 90%B→10%B; 35.1∼45 min, 10%B→10%B) and conditions 3 (0∼2 min, 20%B→20%B; 2∼30 min, 20%B→75%B; 30∼35 min, 75%B→75%B; 35∼35.1 min, 75%B→20%B; 35.1∼45 min, 20%B→20%B), resulting in notable improvement in separation degrees between impurities and between impurities and the main peak on the chromatogram. However, these improvements did not suffice to meet the specified requirements (Figures 2D, E). After several rounds of optimization, the conditions described in Section 2.3 were identified as the definitive chromatographic conditions for the product. These conditions achieved a minimum separation degree of 1.62 among impurities and 1.8 between the impurities and the main peak, thereby meeting the required detection standards.

### 3.2 Method validation

#### 3.2.1 Specificity

The chromatogram of the system suitability solutions was shown in Figure 2F. Results indicated resolutions of 6.23 and 6.93 between the main peak and adjacent impurity peaks, respectively, exceeding the threshold value of 1.5. Additionally, the minimum separation between impurity peaks was 1.75, exceeding the threshold value of 1.2.

**FIGURE 3:**
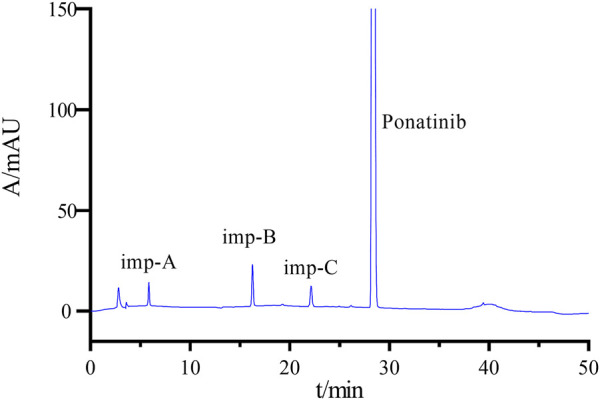
Chromatograms of mixed impurity solutions.

The chromatogram of the mixed impurity solutions was shown in Figure 3. Peaks 1-4 represent imp-A, imp-B, imp-C, and ponatinib in that order. Supplementary Table S1 contains the retention time (RT), relative retention time (RRT), and resolution. The satisfactory separation (>1.5) between these impurities and ponatinib was observed. High-performance liquid chromatography facilitated comprehensive segregation of the associated compounds.

#### 3.2.2 Forced degradation experiments

**FIGURE 4:**
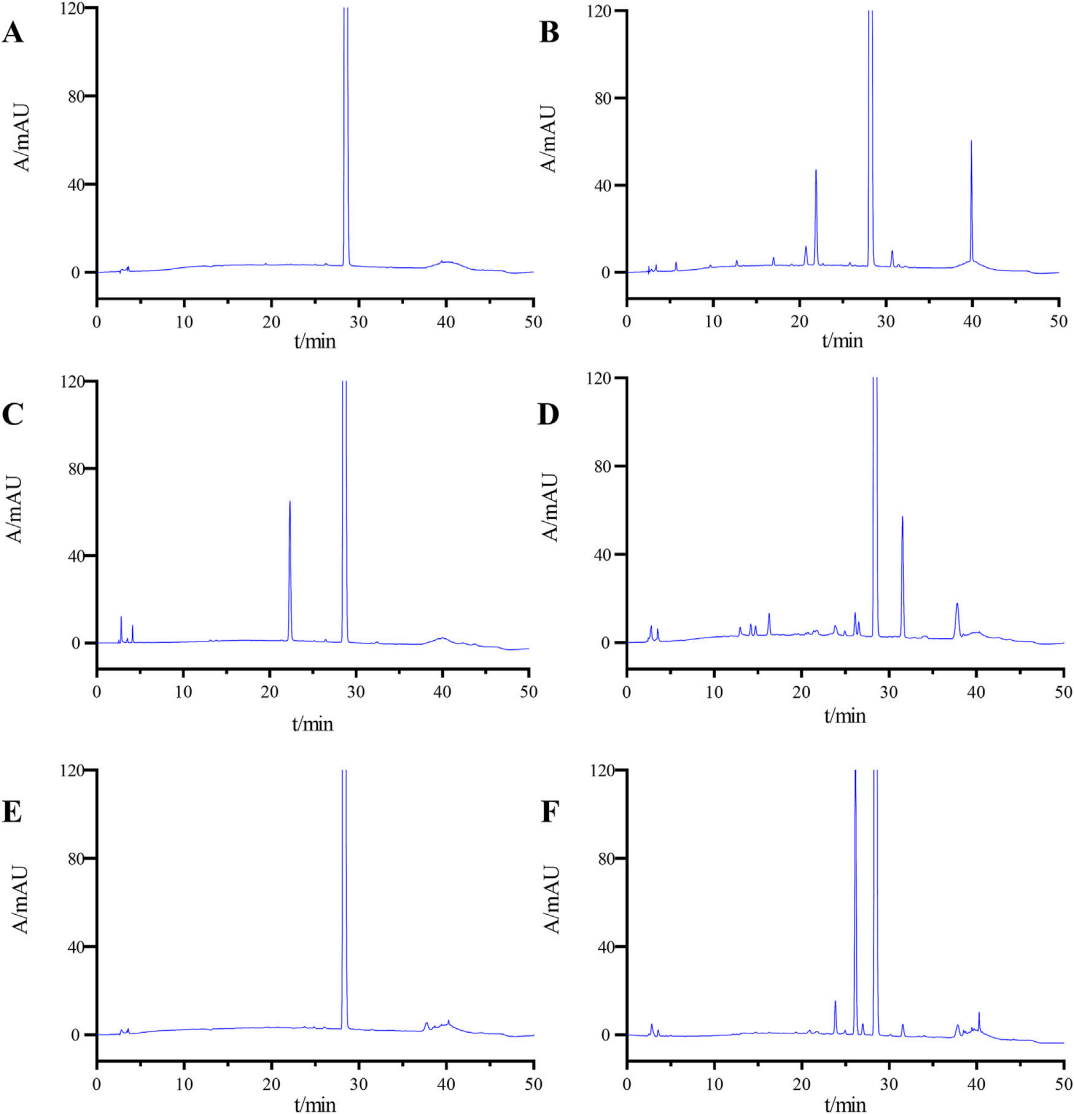
HPLC chromatograms of undegradation **(A)**, acid degradation **(B)**, base degradation **(C)**, oxidative degradation **(D)**, heat degradation **(E)**, and photolytic degradation **(F)**.

Figure 4 presents the chromatographic findings from the forced degradation experiment, and Supplementary Table S2 assesses ponatinib’s stability under these conditions, including impurity levels, main peak content, resolution between principal components and impurities, resolution between impurities, and recovery rate. Ponatinib demonstrates relative stability under high temperature and alkaline conditions but readily degrades under acidic, light and oxidative conditions.

Importantly, under varied degradation conditions, the resolution between the main peak and impurities exceeds 1.5, and between impurities, it surpasses 1.2, meeting the specified criteria. Additionally, the mass balance ranges from 96.5% to 102.1%, indicating a satisfactory material balance from 95% to 105%, a fundamental criterion for equilibrium.

#### 3.2.3 Sensitivity evaluation

The detection limit concentrations of ponatinib and its impurities A, B, and C were determined to be 0.003, 0.002, 0.002, and 0.005 μg/mL, respectively, while the quantitation limit concentrations were 0.008, 0.005, 0.004, and 0.016 μg/mL, respectively. These results clearly demonstrate the high quantitative sensitivity of the newly developed HPLC method for related impurities present in ponatinib at concentrations exceeding 0.05%.

#### 3.2.4 Stability of the solution

Supplementary Table S3 contains the findings from these tests. Furthermore, after exposure to ambient temperature for 24 h, the three parameters (number of impurities, maximum single impurity content, and total impurities content) exhibited negligible changes, confirming the stability of the sample solution at room temperature. A mixed solution containing impurities A, B, and C was prepared and allowed to equilibrate at room temperature for 24 h. Sample detection was conducted at multiple time points, revealing that the relative standard deviations (RSDs) of peak areas for ponatinib and its impurities A, B, and C were 0.85%, 1.29%, 1.72%, and 1.42%, respectively.

#### 3.2.5 Linearity

The regression curves for ponatinib and its impurities are presented in Supplementary Figure S3. Table 1 presents the calibration curves and correlation coefficients. All correlation coefficients within the concentration range from the quantitative limit to 2.0% exceeded 0.99, meeting the experimental requirements.

**TABLE 1:** Linearity of ponatinib and related substances.

Substance	Standard calibration curves	Correlation coefficient (r)
ponatinib	y = 40.53x+1.585	0.9996
imp-A	y = 16.99x+0.7561	0.9995
imp-B	y = 47.24x+0.9415	0.9996
imp-C	y = 29.63x+1.121	0.9994

#### 3.2.6 Precision

For ponatinib and its related substances (imp-A, imp-B, imp-C), the relative standard deviations (RSDs) were 0.05%, 0.07%, 0.12%, and 0.09%, respectively. Similarly, the RSDs for the peak areas were calculated to be 0.88%, 0.67%, 1.23%, and 0.79%. It is important to mention that the entirety of RSDs were less than 2%, which suggests a significant degree of accuracy in the device.

#### 3.2.7 Repeatability

The results indicate good repeatability of the method, with relative standard deviations (RSD) of imp-A, -B, and -C at 1.65%, 1.47%, and 1.71% respectively, all below 2%.

#### 3.2.8 Accuracy

Table 2 presents the recovery rates of known impurities in ponatinib, which range between 90% and 110%. These findings indicate that the method exhibits a high level of accuracy.

**TABLE 2:** Recovery of ponatinib-related substances.

Substance	Target level	Spiked conc. (μg/mL)	Determined conc. (μg/mL)	Recovery (%)	Average recovery rate (%)	RSD (%)
imp-A	50%	0.516	0.521	100.97	104.29	1.66
		0.516	0.551	106.78		
		0.516	0.541	104.84		
	100%	1.032	1.081	104.75		
		1.032	1.064	103.10		
		1.032	1.073	103.97		
	150%	1.548	1.596	103.10		
		1.548	1.637	105.75		
		1.548	1.631	105.36		
imp-B	50%	0.523	0.518	99.04	103.71	3.17
		0.523	0.545	104.21		
		0.523	0.512	97.90		
	100%	1.045	1.117	106.89		
		1.045	1.094	104.69		
		1.045	1.083	103.64		
	150%	1.568	1.677	106.95		
		1.568	1.621	103.38		
		1.568	1.673	106.70		
imp-C	50%	0.524	0.533	101.72	102.48	2.98
		0.524	0.558	106.49		
		0.524	0.511	97.52		
	100%	1.049	1.095	104.39		
		1.049	1.113	106.10		
		1.049	1.096	104.48		
	150%	1.574	1.582	100.51		
		1.574	1.594	101.27		
		1.574	1.571	99.81		

The test conditions have no change compared with that in Table 1.

#### 3.2.9 Robustness

Under acceptable but fluctuating conditions, the resolution between ponatinib and its adjacent impurity peaks exceeds 1.5, while the minimum resolution between other impurity peaks also surpasses 1.2. The number and composition of impurities exhibited stability. Moreover, variations in column temperature, wavelength adjustment, and changes in mobile phase pH did not impact the detection of associated compounds. However, alterations in flow rate, chromatographic column type, and initial mobile phase ratio minimally affected retention time and resolution. Fortunately, minor modifications to these variables exerted a negligible influence on detection outcomes, thereby demonstrating the robustness of this analytical approach. Table 3 provides comprehensive information on the specific requirements for robustness performance.

**TABLE 3:** Test conditions of robustness.

Chromatogram conditions	The variation range of parameters
The initial proportion of mobile phases A-B (%)	86∶14; 84∶16; 82∶18
Wavelength (nm)	245, 250, 255
Column temperature (°C)	35, 40, 45
Flow rate (mL/min)	0.9, 1.0, 1.1
Mobile phase pH	2.2, 2.4, 2.6
Chromatographic column	Waters-C_18,_ Agilent-5HC-C_18,_ Gemini-C_18_

### 3.3 Characterization of imp-B

**FIGURE 5:**
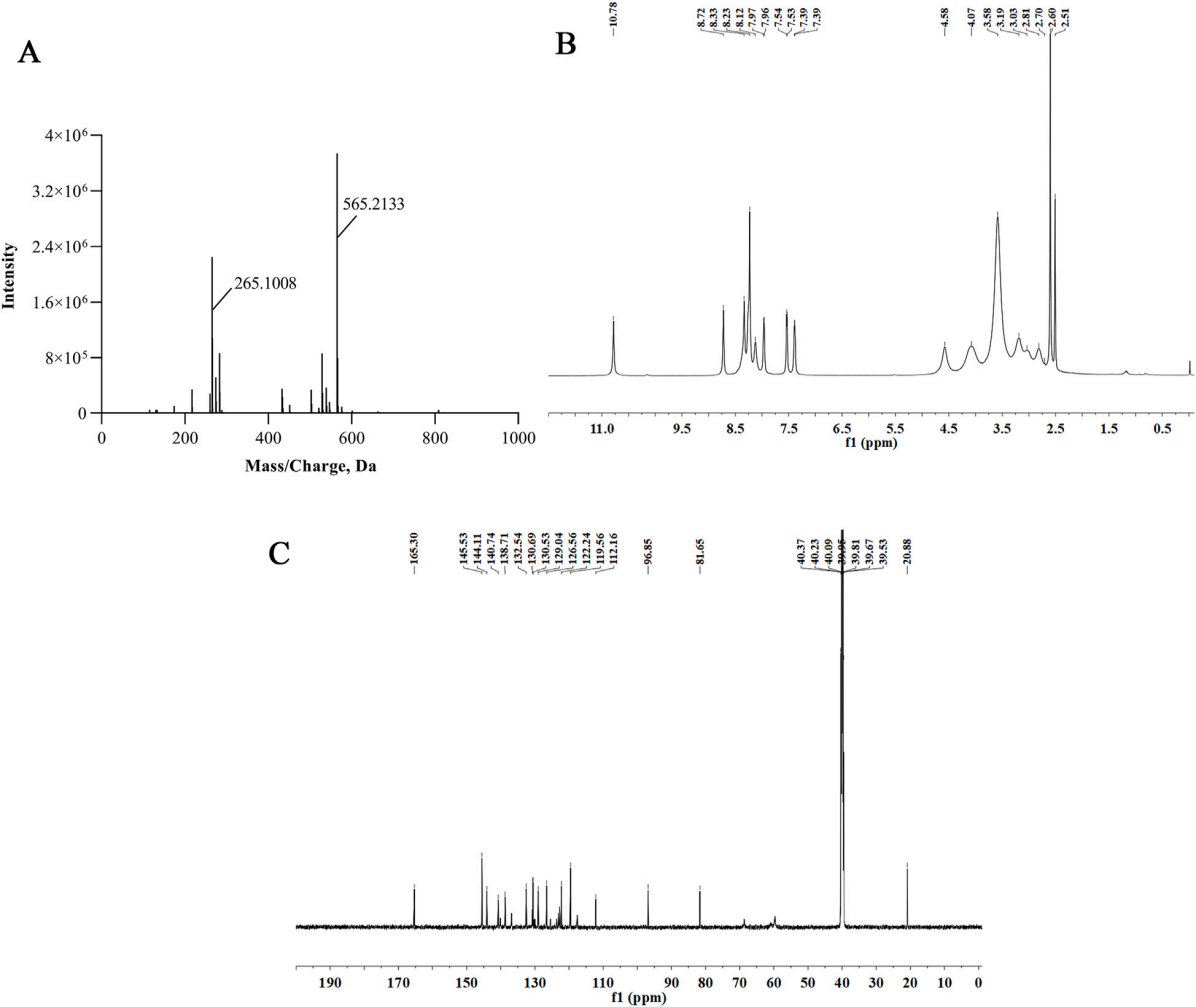
The HRMS **(A)**, ^1^H NMR **(B)** and ^13^C NMR **(C)** spectra of imp-B.

Imp-B was prepared following the procedures outlined in Section 2.4 of the study. The resulting chromatogram in Supplementary Figure S3 showed a retention time of 8.2 min with a purity of 97.5%. Figures 5A–C present the high-resolution mass spectrum, 1D proton nuclear magnetic resonance (NMR) spectrum, and 1D carbon NMR spectrum of imp-B, respectively. Additionally, the 2D NMR spectrum is illustrated in Supplementary Figure S4. Using mass spectrometry and NMR spectroscopy techniques, we successfully elucidated the structure of imp-B (Figure 5A), which possesses a molecular weight of 564. This result was in agreement with previously reported in the literature (Golla et al., 2023).

## 4 Discussion

Ponatinib, a third-generation tyrosine kinase inhibitor (TKI), is utilized in the treatment of chronic myeloid leukemia and acute lymphoblastic leukemia associated with specific gene mutations (Kantarjian et al., 2022). The related substances in ponatinib, including process impurities and degradation impurities, represent critical factors influencing its safety and efficacy. Golla, V. M. et al. investigated the degradation impurities of ponatinib through degradation testing (Golla et al., 2023); however, they did not examine the process impurities generated during its synthesis. Currently, no method exists that can simultaneously detect both process impurities and degradation impurities in ponatinib. Consequently, the objective of this study is to develop a novel method capable of simultaneously detecting both process impurities and degradation impurities in ponatinib.

HPLC technology has emerged as a preferred separation technique for determining active ingredients and related substances in pharmaceutical samples, owing to its convenience, simplicity, stability, and cost-effectiveness (Zhao, et al., 2022). Furthermore, the majority of bulk drugs listed in the United States Pharmacopeia (USP) and the European Pharmacopeia (EP) utilize HPLC to detect related substances. Nevertheless, HPLC technology possesses certain limitations, including challenges in detecting impurities at very low concentrations and in separating specific chiral isomers.

Based on the synthetic process route and degradation experiments of ponatinib, we investigated its related substances. The investigation revealed that imp-A is a process impurity, imp-C serves as both a process impurity and an alkaline degradation impurity, and imp-B is formed through oxidative degradation. Structural analysis using NMR and HRMS revealed that imp-B is a previously unreported compound. While various degradation products were observed in rigorous degradation experiments, effective isolation was challenging due to either their low concentrations or their close association with adjacent components. Therefore, this study did not extensively investigate these additional degradation products, indicating the need for further exploration in future studies.

To establish a methodology for identifying related substances in Ponatinib, this study initially prepared a system suitability solution encompassing a wide array of process impurities and degradation products. The detection method was subsequently refined by investigating the effects of solvent selection, detection wavelength, mobile phase composition, and elution technique on the separation of related substances.

The developed method underwent validation following the guidelines of the International Council for Harmonisation of Technical Requirements for Registration of Pharmaceuticals for Human Use (ICH) Guideline Q2 (R1) (Group, 2005). The results demonstrated satisfactory performance in terms of specificity, sensitivity, solution stability, linearity, precision, accuracy, and robustness.

## 5 Conclusion

In this study, we developed a productive HPLC approach to detect compounds associated with ponatinib used in the treatment of CML. Furthermore, we successfully validated this detection methodology. The results demonstrate remarkable specificity, enhanced sensitivity, and satisfactory linearity, precision, repeatability, and robustness. In this study, we developed a robust HPLC method to detect compounds associated with ponatinib used in the treatment of chronic myeloid leukemia (CML). Furthermore, we successfully validated this detection methodology. The results demonstrate exceptional specificity, increased sensitivity, and satisfactory linearity, precision, repeatability, and robustness. Therefore, this study will serve as a valuable reference for the treatment of CML with ponatinib.

## Data Availability

The original contributions presented in the study are included in the article/Supplementary Material, further inquiries can be directed to the corresponding authors.
