# Supplementary material for: Chromatographic analysis of ponatinib and its impurities: method development, validation, and identification of new degradation product
Source: Front Chem. 2024 Nov 12;12:1487108. doi: 10.3389/fchem.2024.1487108 (PMC11588477; doi:10.3389/fchem.2024.1487108)
Supplement: Supplementary file 1 [file DataSheet1.docx]

Supplementary Material


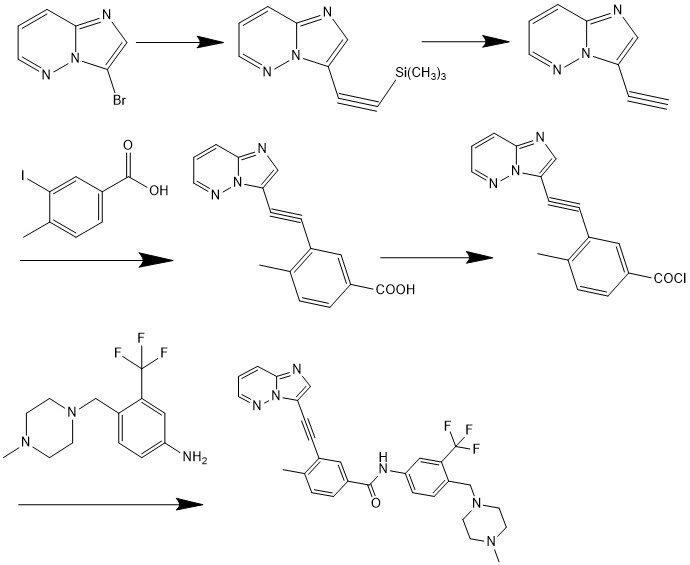


**Figure S1** Synthetic route of ponatinib


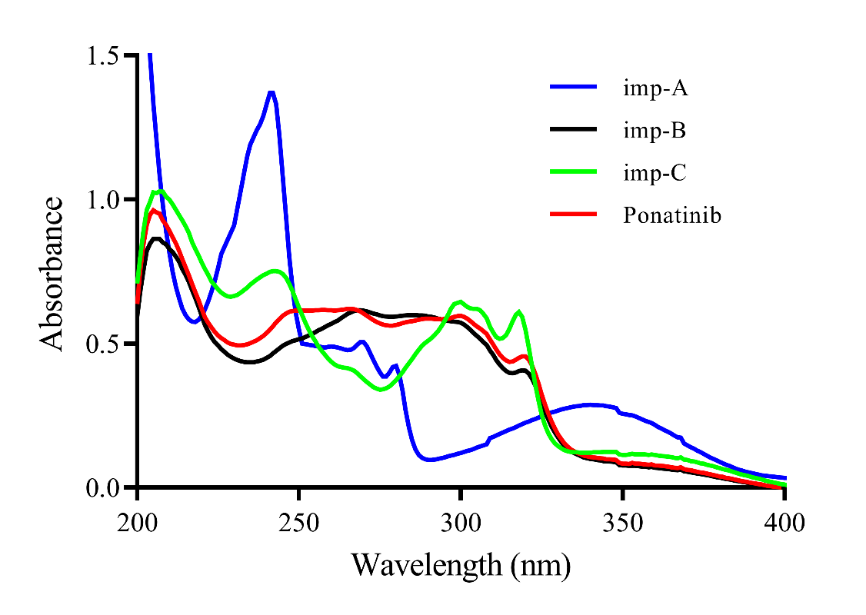


**Figure S2** Ultraviolet spectrogram of ponatinib and related substances

**
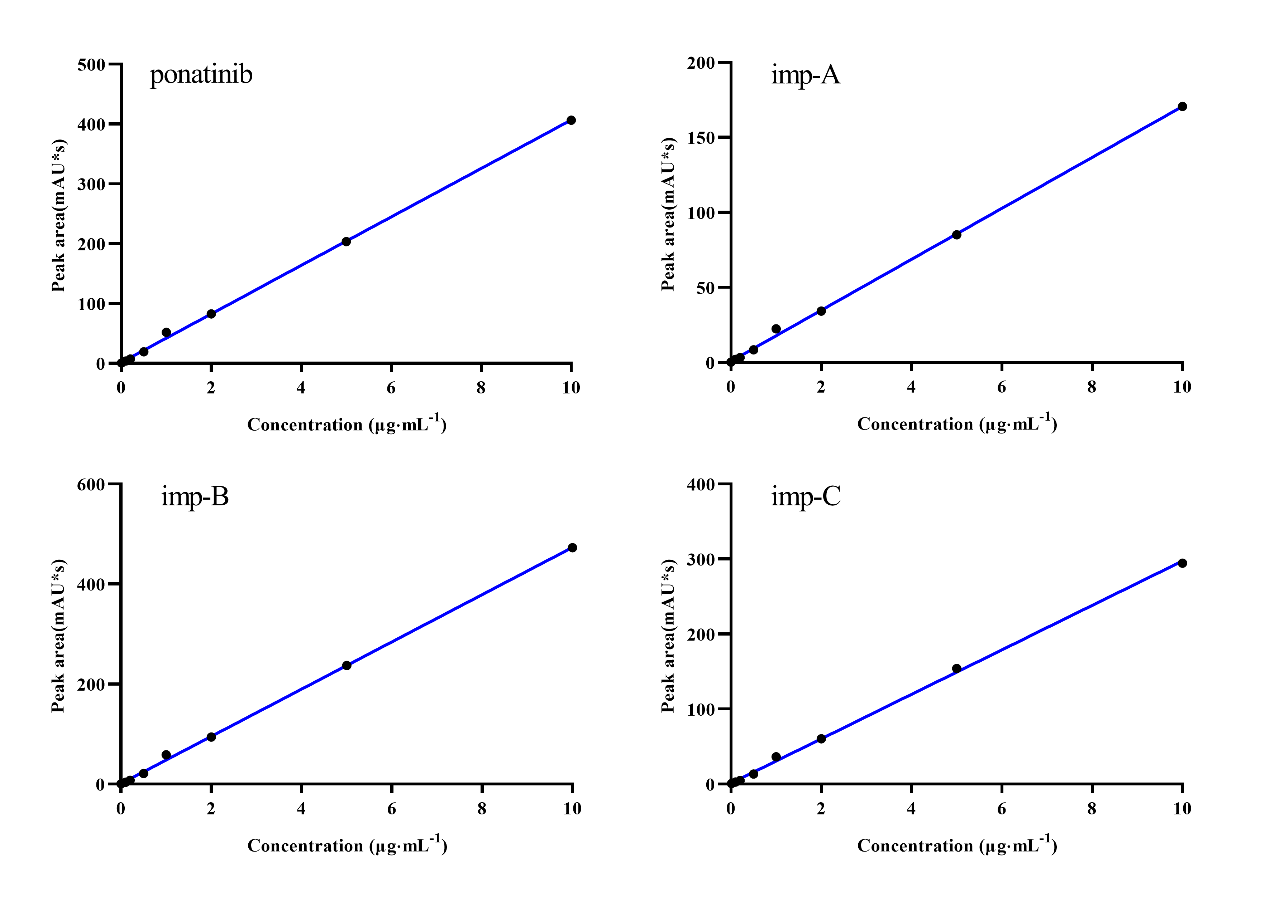
**

**Figure S3** Regression curves of ponatinib and related substances


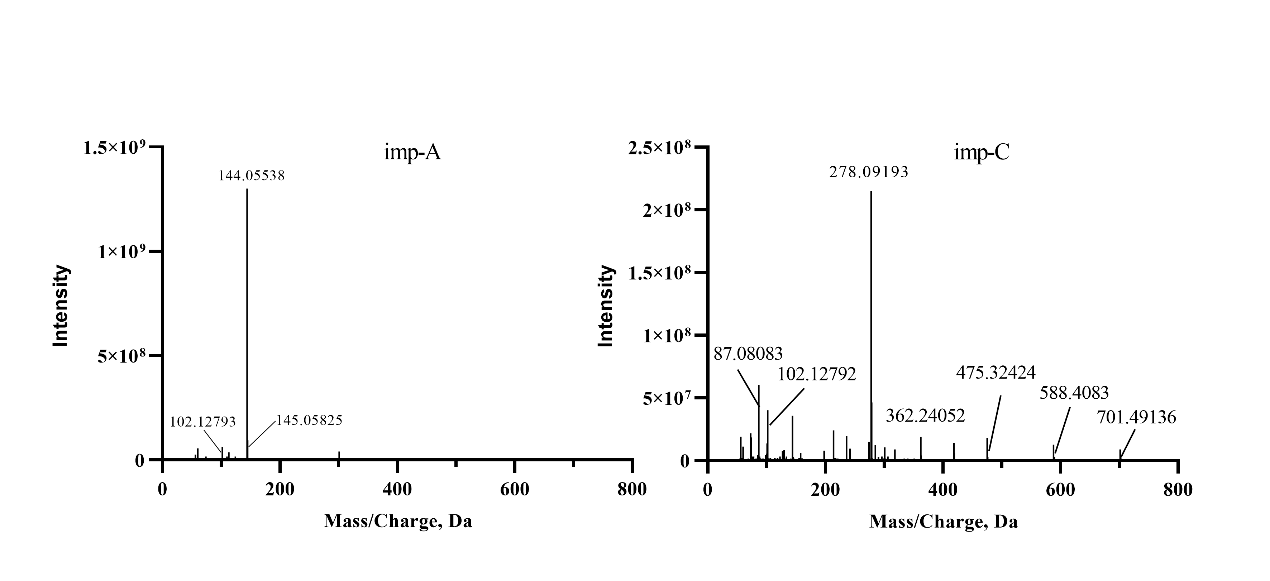


**Figure S4** The high-resolution mass spectrometry graphs of imp-A and imp-C

**Table S1** Results of separation tests for ponatinib and related substances

| **Peak** | **Name** | **Retention time (min)** | **Relative retention time** | **Resolution** |
| --- | --- | --- | --- | --- |
| 1 | imp-A | 5.862 | 0.21 | 9.18 |
| 2 | imp-B | 16.268 | 0.58 | 42.42 |
| 4 | imp-C | 22.150 | 0.78 | 19.97 |
| 5 | ponatinib | 28.277 | 1.00 | 5.71 |

Test conditions: column, an Agilent 5HC-C_18_ column (4.6 mm×250 mm, 5 μm); detection wavelength, 250 nm; flow rate, 1.0 mL/min; injection volume, 10 µL; mobile phase A, aqueous solution and ACN (9:1, v/v) containing 2 mM KH_2_PO_4_ and 0.4% triethylamine (pH 2.4); mobile phase B, ACN.

**Table S2** Results of forced degradation tests

| **Forced Degradation Condition** | **Number of impurities（>0.03 %）** | **Content of main peak (%)** | **Minimum resolution between main peak and impurities** | **Minimum resolution among impurities** | **Mass balance (%)** |
| --- | --- | --- | --- | --- | --- |
| Undegradation | 3 | 99.86 | 4.84 | 3.26 | 100 |
| Acid degradation | 13 | 92.90 | 6.32 | 1.57 | 96.5 |
| Base degradation | 6 | 94.31 | 5.78 | 5.14 | 97.3 |
| Oxidative degradation | 12 | 91.45 | 5.07 | 1.44 | 98.3 |
| Heat degradation | 5 | 99.29 | 5.65 | 3.82 | 102.1 |
| Photolytic degradation | 9 | 88.91 | 5.51 | 1.78 | 98.1 |

**Table S3** Test results of ponatinib sample solution stability

| **Time/h** | **Number of impurities** | **Content of maximum single impurity (%)** | **Content of total impurities (%)** |
| --- | --- | --- | --- |
| 0 | 3 | 0.06 | 0.14 |
| 1 | 3 | 0.05 | 0.14 |
| 2 | 3 | 0.06 | 0.15 |
| 4 | 3 | 0.05 | 0.14 |
| 6 | 3 | 0.07 | 0.16 |
| 8 | 3 | 0.06 | 0.15 |
| 12 | 3 | 0.07 | 0.16 |
| 24 | 3 | 0.08 | 0.17 |
